# Development and validation of a pathomics-driven machine learning model for individualized prediction of neoadjuvant chemotherapy response and early recurrence in HR-positive, HER2-negative breast cancer

**DOI:** 10.3389/fonc.2026.1770037

**Published:** 2026-02-23

**Authors:** Jiaxian Yue, Jiaxiang Liu, Xiyu Kang, Pei Yuan, Wei Wang, Zhanyu Wang, Chao Shang, Qingyao Shang, Guangyu Li, Xubin Dong, Tianxiao Wang, Dongmin Yang, Shuhao Wang, Chenxuan Yang, Jianming Ying, Xin Wang

**Affiliations:** 1 Department of Breast Surgical Oncology, National Cancer Center/National Clinical Research Center for Cancer/Cancer Hospital, Chinese Academy of Medical Sciences and Peking Union Medical College, Beijing, China; 2 Beijing Tsinghua Changgung Hospital, School of Clinical Medicine, Tsinghua University, Beijing, China; 3 Department of Pathology, National Cancer Center/National Clinical Research Center for Cancer/Cancer Hospital, Chinese Academy of Medical Sciences and Peking Union Medical College, Beijing, China; 4 Thorough Lab, Thorough Future, Beijing, China

**Keywords:** artificial intelligence, breast cancer, HR-positive/HER2-Negative, machine learning, neoadjuvant chemotherapy, pathomics, prediction

## Abstract

**Background:**

Hormone receptor (HR)-positive, human epidermal growth factor receptor 2 (HER2)-negative breast cancer is the most prevalent subtype among women but has a modest response to neoadjuvant chemotherapy (NAC). Accurately predicting NAC efficacy and recurrence risk remains challenging, as conventional clinical and molecular markers have limited predictive power. Advances in digital pathology and artificial intelligence now enable quantitative pathomics analysis, offering new opportunities for precise prediction and prognostic assessment.

**Methods:**

In this retrospective study, 162 HR-positive, HER2-negative breast cancer patients treated with NAC between 2014 and 2021 were included. Hematoxylin and eosin (H&E)-stained pretreatment biopsy slides were digitized and analyzed using Vision Transformer (ViT) and Unified Network for Image (UNI) deep learning models to extract pathomic features. Thirteen clinical variables were collected. After least absolute shrinkage and selection operator (LASSO)-based feature selection, multiple machine learning models were developed for both response prediction and prognostic evaluation of recurrence, with performance evaluated by receiver operating characteristic (ROC) curves, area under the curve (AUC), sensitivity, specificity, confusion matrix, calibration curves, and decision curve analysis (DCA). Furthermore, SHapley Additive exPlanations (SHAP) was used to rank the importance of features for each model.

**Results:**

The CatBoost model achieved the best predictive performance (AUC = 0.900 in training and 0.848 in validation) when a combination of clinical and pathomics-derived variables was used. Key predictive factors included Ki-67 expression, age, histological grade, PR status, and prominent pathomic features. A Kaplan–Meier survival plot indicated that regardless of stratification by MP grade or pCR status, there was no significant difference in recurrence status or survival outcomes between the two groups in this cohort. Furthermore, the recurrence models developed mainly using pathomics were strongly accurate for predicting 1-year recurrence (AUC = 0.907 in training and 0.769 in validation).

**Conclusions:**

Integrating pathomic features with clinical variables via machine learning enables robust pretreatment prediction of NAC efficacy and short-term recurrence in HR-positive, HER2-negative breast cancer. This approach has the potential to offer a clinically practical tool to optimize individualized therapy and improve patient management, highlighting the translational value of AI-powered digital pathology in breast cancer care.

## 1 Introduction

Breast cancer is among the most common malignant tumors among women, and as its incidence has continued to rise in recent years, it has become a leading cause of cancer-related mortality in women worldwide (1, 2). Among the molecular subtypes of breast cancer, the hormone receptor (HR)-positive, human epidermal growth factor receptor 2 (HER2)-negative subtype accounts for approximately 60–70% of all cases and represents the most prevalent form of the disease (3). Although this subtype of breast cancer generally exhibits relatively indolent biological behavior and favorable long-term prognosis, a proportion of patients experience recurrence and progression during treatment. Optimizing treatment strategies and accurately predicting treatment response and prognosis in this population remain significant challenges in clinical management.

Neoadjuvant chemotherapy (NAC), a crucial component of comprehensive breast cancer management, can reduce tumor volume prior to surgery, thereby facilitating surgical intervention, increasing the breast-conserving rate, and providing a window for assessing tumor biological response (4, 5). However, patients with HR-positive, HER2-negative breast cancer generally exhibit a low pathological complete response (pCR) rate to NAC, with marked interindividual heterogeneity (6, 7). Consequently, some patients are subjected to the toxicity of chemotherapy without benefitting from NAC, and they may even miss the optimal timing for surgery. Although previous studies have explored the predictive value of clinical parameters and conventional molecular pathological markers, there remains a lack of effective tools in current clinical practice to accurately predict NAC efficacy for this subtype prior to treatment (8, 9). In addition, the survival benefit associated with pCR in this subtype remains controversial (10, 11). Therefore, accurately identifying the high-risk population for recurrence after NAC and promptly adjusting therapeutic strategies are critical for maximizing patient benefit and improving survival outcomes.

Recent advances in digital pathology and artificial intelligence (AI) technologies have provided new approaches for addressing the aforementioned challenges. Pathomics, an emerging research methodology that enables high-throughput, automated, and quantitative feature extraction from digitized pathological slides, has been increasingly refined. This technique objectively and comprehensively reveals the histological heterogeneity and complexity of tumors, demonstrating promising applications in cancer diagnosis, prognostic assessment, and treatment response prediction (12–14). Concurrently, machine learning (ML) algorithms, which simulate human behavior to enable intelligent learning and data processing, have become powerful tools for assisting in disease diagnosis and treatment (15).

In recent years, several studies have attempted to predict the efficacy of NAC in breast cancer by constructing models based on pathomics feature extraction combined with ML approaches. For example, li et al. (16) developed a pathomics-based model to predict the Residual Cancer Burden (RCB) score in breast cancer patients receiving NAC, while Fisher et al. (12)explored the role of ML integrated with digital pathology image analysis in predicting the response to NAC in triple-negative breast cancer. However, these studies generally did not focus on the HR-positive, HER2-negative subgroup, which is less sensitive to chemotherapy, and mostly targeted a single endpoint (such as pCR), rarely addressing early relapse risk, an equally critical factor for clinical decision-making, thereby limiting the models’ utility in personalized treatment and follow-up management.

In this context, in the present study, pathomic features with clinical data and multiple machine learning (ML) algorithms were used to develop a predictive model for the efficacy of neoadjuvant chemotherapy (NAC) in patients with HR-positive, HER2-negative breast cancer. Furthermore, the relationship between NAC response and patient prognosis was systematically analyzed, and the value of pathomic information in prognosis prediction was explored. By applying rigorous and reproducible modeling and validation procedures, the aim of this study was to provide an accurate and clinically practical tool for predicting therapeutic response and recurrence risk. Such a tool has the potential to optimize treatment decision-making and advance the implementation of precision therapy for this breast cancer subtype.

## 2 Methods

### 2.1 Patient selection

**Figure 1:**
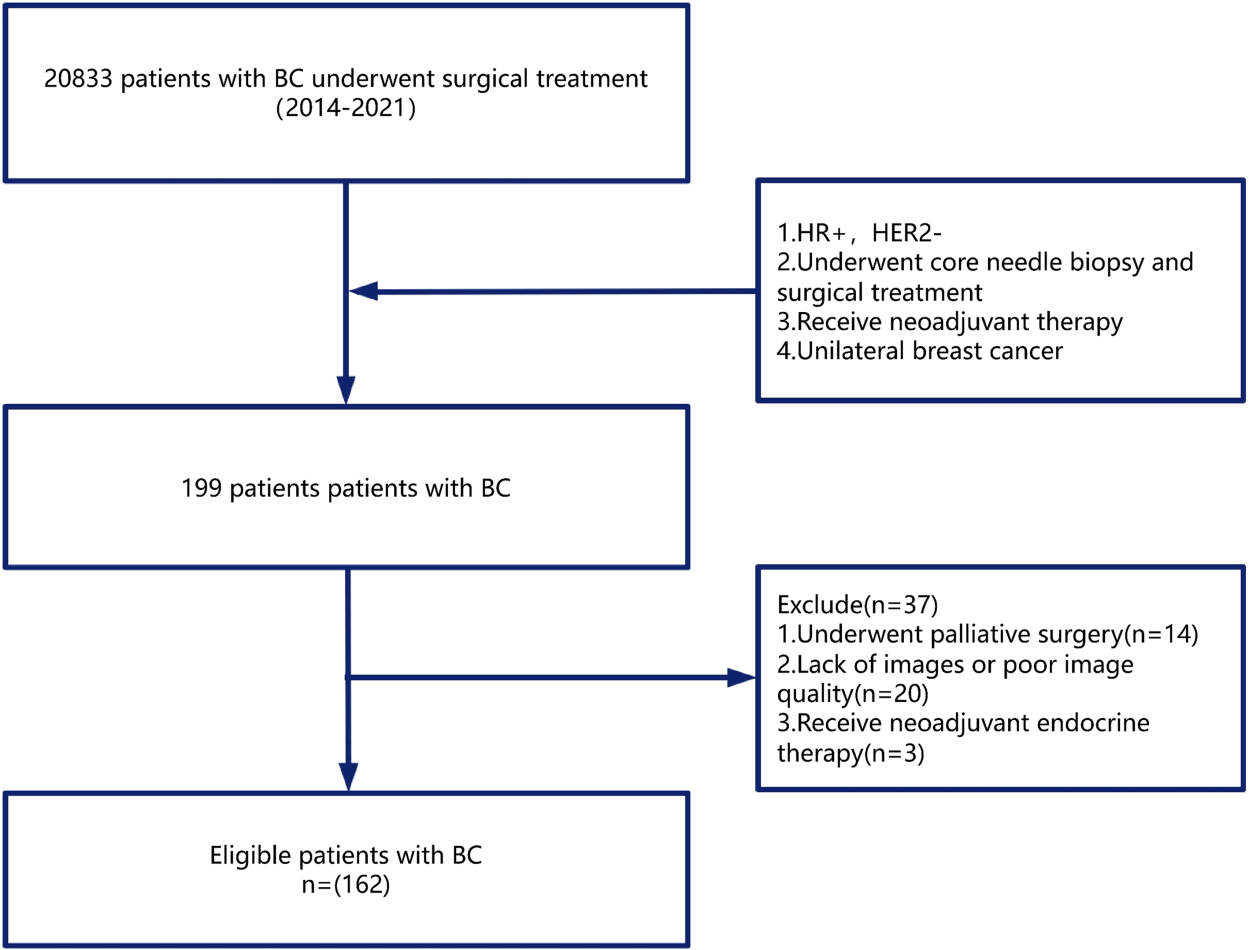
Flow diagram of patient cohort selection.

In this retrospective study, patients who were diagnosed with breast cancer between Jan. 2014 and Dec. 2021 at the Cancer Hospital Chinese Academy of Medical Sciences were included (**Figure 1**). The inclusion criteria were as follows: female patients (1) who were diagnosed with primary breast cancer by pathological examination (2), who underwent pre-NAC core needle biopsy and surgical treatment (3), whose biopsy pathology confirmed that they were hormone receptor-positive and HER2-negative, and (4) who underwent neoadjuvant therapy before surgery. The exclusion criteria were as follows (1): patients who were diagnosed with bilateral breast cancer (2), patients who underwent palliative surgery (3), patients who lacked biopsy H&E-stained histopathological slide scan images or whose scan images were of low quality, and (4) patients who underwent neoadjuvant endocrine therapy. Patient information regarding age; clinical stage; tumor grade; ER, PR, and Ki67 levels; neoadjuvant chemotherapy regimen; surgical approach; postoperative pathological stage; height; weight; menopausal status; family history; tumor position; and metabolic indicators, including blood glucose, TG, and LDL-C levels, was collected from the medical records. Patients were followed up for a median duration of 66 months.

### 2.2 Pathology slide scanning and feature extraction

On the basis of Vision Transformer (ViT) and Unified Network for Image (UNI) pretrained models, an integrated framework was constructed in this study to enable efficient and precise feature extraction from pathological slides. The workflow comprised the following main stages:

#### 2.2.1 Input and image patching

Whole-slide images (WSIs) were obtained by scanning H&E-stained histopathological slides from pre-neoadjuvant core needle biopsy specimens. To facilitate model processing, each WSI was divided into multiple image patches of size 224×224 pixels as preprocessing. Each patch was independently processed for feature extraction in the subsequent steps.

Each 224×224 patch was subdivided into nonoverlapping 16×16 subpatches, which were then flattened into a sequence of patch tokens. Afterward, each patch token was linearly projected into a fixed-dimensional vector space, resulting in a set of patch embeddings. After linear projection, the input feature dimension was [*B*, *N*, *D*], where B denotes the batch size (set to 2 in this study), N is the number of tokens per image ( N=(224/16)×(224/16)=196), and D is the embedding dimension ( D=1024). An additional class token (CLS token) was added to the sequence of the patch embeddings to aggregate the global information from the entire image.

#### 2.2.2 Transformer encoder

The patch embeddings, including the CLS token, were input into a transformer encoder. Through the multihead self-attention mechanism, the encoder modeled global interactions among all the patches, thereby capturing long-range dependencies and contextual information. The output of the transformer encoder maintained the dimensionality [*B*, *N*, *D*], meaning that each patch token was represented by a contextually enhanced feature vector.

#### 2.2.3 Global feature aggregation (Global pooling)

The output of the transformer encoder underwent global average pooling, aggregating the feature vectors from all the patch tokens to generate a global semantic representation of the entire image. The resulting feature dimension was [*B*, *D*], indicating that each image was encoded as a 1024-dimensional feature vector. This feature vector was subsequently saved for downstream analyses or further processing.

### 2.3 Prediction model construction and validation

The construction and validation process of the prediction model is illustrated in **Supplementary Figure S1**. The detailed steps are as follows:

#### 2.3.1 Construction of training and validation sets with feature selection via LASSO regression

The model construction and validation processes were conducted with R 4.3.1. A total of 162 patients were randomly divided into a training set and a validation set in a 70% to 30% ratio. Given the relatively low number of positive events, stratified splitting was employed to ensure balanced event distribution. For patients with multiple slides, the mean of each pathomic feature across slides was calculated to construct models at the patient level.

Least absolute shrinkage and selection operator (LASSO) regression was applied to both clinical variables and the previously extracted 1024-dimensional pathomic features to identify variables for subsequent model construction (clinical variables excluded any postneoadjuvant information such as post-operative pathological stage). Prior to lasso regression, variables with zero standard deviation (SD = 0) were removed to eliminate noninformative features and reduce noise and computational burden. After the above data preprocessing steps, a 10-fold cross-validation approach (with the number of folds reduced to as few as 2 if the number of positive events was insufficient) was used. The optimal regularization parameter λ was chosen on the basis of an “acceptable AUC loss threshold”, balancing model performance with the retention of potential predictive variables. This feature selection process was conducted exclusively within the training set.

#### 2.3.2 Hyperparameter optimization

To determine the optimal hyperparameters for each model, 5-fold cross-validation combined with a global random search strategy was employed. The training set was randomly divided into five folds; for each iteration, four folds were used for model training with randomly selected hyperparameters, and the remaining fold served as the validation set to calculate the area under the curve (AUC) and Brier score. The process was repeated until each fold served as the validation set. The scoring metric was calculated as follows: *Score*=mean(AUC)−*α*×mean(Brier),(*α* = 0.5). This process was repeated 50 times, and the hyperparameter set with the highest score was selected for subsequent model construction.

#### 2.3.3 Out-of-fold estimation for optimal tree number and model evaluation on the training set

To avoid optimism bias, out-of-fold (OOF) prediction (F = 5) was used to accurately assess model performance on the training set. To control model complexity, the outer training subset in each OOF split was divided into inner folds, and a 5-fold cross-validation was conducted to estimate the optimal number of iterations and train the model. Early stopping was applied during inner fold training, and the “median” number of best-trees from all folds was used as the robust number of iterations.

To increase the stability and predictive performance, a weighted ensemble model comprising five submodels was constructed: Each submodel randomly sampled a feature subset (80–100% of the features were used). For each submodel, the mean AUC from the inner folds was recorded as the ensemble weight, and the median best-trees served as the fixed iteration count. Each submodel was retrained on the outer training subset using this fixed iteration number. Predictions were generated for the outer validation subset, and the mean AUC from the inner folds was used as the ensemble weight to aggregate the OOF probabilities, which represented the final training set predictions for each algorithm.

#### 2.3.4 Platt calibration of OOF predictions

To align model-predicted probabilities with true event rates for reliable Brier scores and threshold-based decision-making, Platt calibration was performed on the OOF probabilities. The calibration function was defined as $p_{cal}=\sigma (a+b\cdot \text{logit}(p))$, where σ is the sigmoid function.

#### 2.3.5 Retraining on the complete training set and validation

The final model was trained using the entire training set. For each submodel, a new feature subset was sampled. Inner folds of the complete training set were used to determine the mean AUC (ensemble weight) and median tree number (fixed iteration count). Each submodel was retrained on the full training set with a fixed iteration count, and all five submodels were saved. The ensemble model was then evaluated on the independent validation set to generate raw probability predictions, which were subsequently calibrated using the Platt calibrator fitted on the OOF results, providing the final probability estimates on the validation set.

#### 2.3.6 Model evaluation

On the basis of the above training strategy, predictive models were constructed using five machine learning algorithms: CatBoost, random forest, LightGBM, XGBoost, and support vector machine (SVM). Model performance was assessed using receiver operating characteristic (ROC) curves, area under the curve (AUC), sensitivity, specificity, confusion matrix, calibration curves, and decision curve analysis (DCA). SHapley Additive exPlanations (SHAP) was used to rank the importance of features for each model.

### 2.4 Statistics

All the statistical analyses in this study were performed using R 4.3.1. The main analytical workflow included the description and comparison of baseline characteristics and clinical variables between the two groups, Kaplan–Meier survival analyses with log-rank tests, and both univariable and multivariable Cox proportional hazards regression modeling. Unless otherwise specified, all the statistical tests were two-sided, and the significance threshold was set at P<0.05. For comparisons of continuous variables between groups, the Shapiro–Wilk test was first used within each group to assess the normality of the distribution. If both groups exhibited an approximately normal distribution, the Welch t test (not assuming equal variances) was applied; otherwise, the Wilcoxon rank-sum test (Mann–Whitney U test) was used. For categorical variables, the Pearson chi-square test was used preferentially; when any expected cell count was less than 5 or the chi-square test could not be computed, Fisher’s exact test was employed. To compare the prognostic outcomes between the two groups, a Kaplan–Meier survival analysis was conducted according to the predefined grouping variable, and survival curves were plotted with P values derived from the log-rank test. Risk tables and reference lines for median survival are also displayed. To quantify the associations between each candidate variable and overall survival (OS) and disease-free survival (DFS), univariable and multivariable Cox proportional hazards regression analyses were performed, and the hazard ratio (HR) and its 95% confidence interval (CI) were reported. Candidate variables included age, cT, cN, surgical approach, ER, PR, Ki-67, grade, chemotherapy regimen, ypT stage, ypN stage, menopausal status, family history, BMI, LDL-C level, TG level, glucose level, MP grade, and pCR. Considering that the MPgrade and pCR may act as mediators of the effects of other variables on prognosis, these two variables were not included as adjustment factors when multivariable Cox proportional hazards models were constructed. For each candidate variable other than MPgrade or pCR, all the variables except MPgrade, pCR, and the variable under analysis itself were included as covariates (to avoid adjusting for the variable being analyzed).

### 2.5 Ethics approval and consent to participate

All patient-related studies were approved by the Ethics Committee of National Cancer Center/Cancer Hospital, Chinese Academy of Medical Sciences and Peking Union Medical College(24/496-4776). All enrolled patients provided informed written consent.

## 3 Results

### 3.1 Baseline characteristics of the enrolled patients

**Table 1** summarizes the baseline characteristics of all the patients in the study. A total of 162 breast cancer patients were enrolled, all of whom were HR-positive, HER2-negative and received neoadjuvant chemotherapy (NAC) (**Figure 1**). The Miller–Payne (MP) grading system was used to evaluate postoperative H&E-stained histopathological slides, and patients were stratified into a response group and a nonresponse group according to MP grade. The response group comprised 34 patients (21%) with MP grades 4 (a marked reduction in invasive tumor cells by more than 90%) or 5 (no invasive tumor cells remaining in the tumor bed) (17), whereas the nonresponse group included 128 patients (79%) with MP grades 1, 2, or 3. There were significant differences between the two groups in terms of age at diagnosis, menopausal status, surgical approach, estrogen receptor (ER) expression, progesterone receptor (PR) expression, Ki-67 index, and histological grade (all P<0.05). Specifically, compared with patients in the nonresponse group, patients in the response group were younger at diagnosis (43.65 vs. 52.02 years), with a greater proportion of premenopausal women (73.5% vs. 52.3%). Moreover, the rate of breast-conserving surgery (BCS) was greater in the response group (20.6% vs. 4.7%), reflecting the impact of NAC efficacy on surgical strategy. In terms of tumor biological features, the response group had lower ER (0.46 vs. 0.81) and PR (0.33 vs. 0.55) expression but a higher Ki-67 index (0.49 vs. 0.27) and higher histological grade than the nonresponse group did, indicating that poorly differentiated and highly proliferative tumors—biologically more similar to the triple-negative phenotype, despite being HR-positive, HER2-negative—may be more sensitive to chemotherapy. No significant differences in other clinical variables, including tumor stage, chemotherapy regimen, body mass index (BMI), family history, or metabolic parameters, were detected between the two groups (**Table 1**).

**Table 1:** Clinical characteristics of patients.

Characteristics	Category	Nonresponse (n=128)	Response (n=34)	P value
Age		52.02 ± 11.68	43.65 ± 10.63	<0.001
Clinical Stage	II	30 (23.4%)	9 (26.5%)	0.887
	III	98 (76.6%)	25 (73.5%)	
cT	1	14 (10.9%)	2 (5.9%)	0.397
	2	65 (50.8%)	22 (64.7%)	
	3	16 (12.5%)	5 (14.7%)	
	4	33 (25.8%)	5 (14.7%)	
cN	0	12 (9.4%)	1 (2.9%)	0.626
	1	34 (26.6%)	9 (26.5%)	
	2	45 (35.2%)	15 (44.1%)	
	3	37 (28.9%)	9 (26.5%)	
Surgery	BCS	6 (4.7%)	7 (20.6%)	0.007
	Mastectomy	122 (95.3%)	27 (79.4%)	
ER		0.81 ± 0.19	0.46 ± 0.39	<0.001
PR		0.55 ± 0.31	0.33 ± 0.31	<0.001
KI67		0.27 ± 0.14	0.49 ± 0.28	<0.001
Grade	I	9 (7.0%)	0 (0.0%)	<0.001
	II	105 (82.0%)	21 (61.8%)	
	III	14 (10.9%)	13 (38.2%)	
Tumor Position	L	66 (51.6%)	18 (52.9%)	1.000
	R	62 (48.4%)	16 (47.1%)	
Chemotherapy Regimen	AT-based	105 (82.0%)	27 (79.4%)	0.727
	A-based	3 (2.3%)	0 (0.0%)	
	T-based	20 (15.6%)	7 (20.6%)	
Height		159.39 ± 5.67	159.62 ± 4.88	0.817
Weight		62.86 ± 10.20	60.24 ± 8.96	0.147
BMI		24.77 ± 4.13	23.60 ± 2.98	0.201
Menopausal Status	NO	67 (52.3%)	25 (73.5%)	0.043
	YES	61 (47.7%)	9 (26.5%)	
Family History	NO	92 (71.9%)	20 (58.8%)	0.209
	YES	36 (28.1%)	14 (41.2%)	
LDL-C		3.12 ± 0.86	2.90 ± 0.94	0.204
TG		1.44 ± 1.01	1.18 ± 0.66	0.148
Glucose		5.78 ± 1.67	5.65 ± 1.59	0.263

### 3.2 Construction and validation of the predictive model for neoadjuvant chemotherapy response

**Figure 2:**
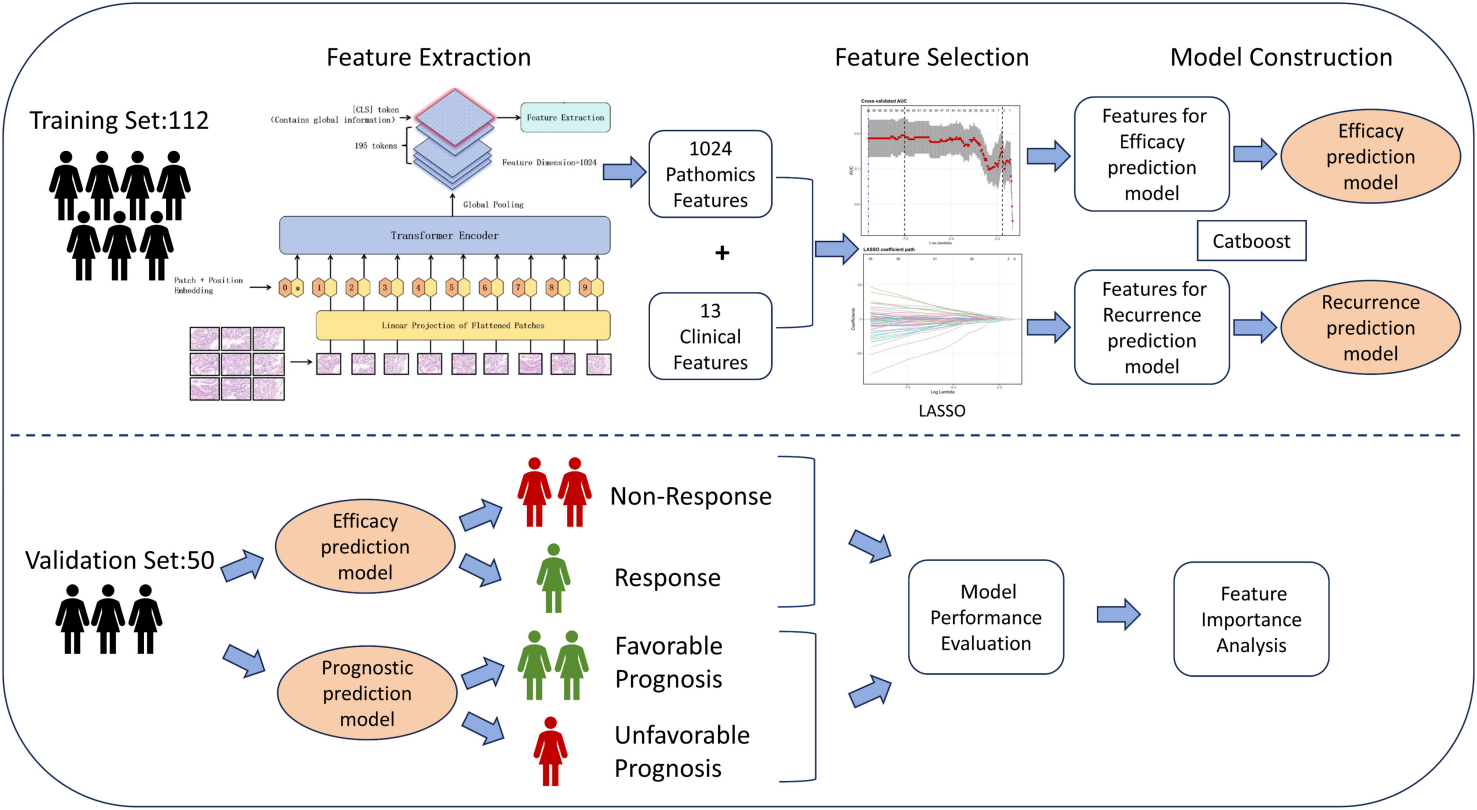
Workflow of study. The clinical information of 162 HR+/HER2- breast cancer patients and HE-stained pathological slide images from core needle biopsies before neoadjuvant chemotherapy were retrospectively collected. The ViT and UNI pretrained models were used to extract features, ultimately yielding 1024 pathological features. These features were combined with 13 collected clinical information features, and LASSO regression was used for feature selection. Selected features were subsequently obtained for efficacy prediction and the construction of the prognostic model. The validation and performance evaluation of the models were subsequently performed on the validation set, and the importance ranking of the features included in the models was determined. HE, hematoxylin and eosin; ViT, Vision Trans-former; UNI, Unified Network for Image; LASSO, least absolute shrinkage and selection operator.

**Figure 3:**
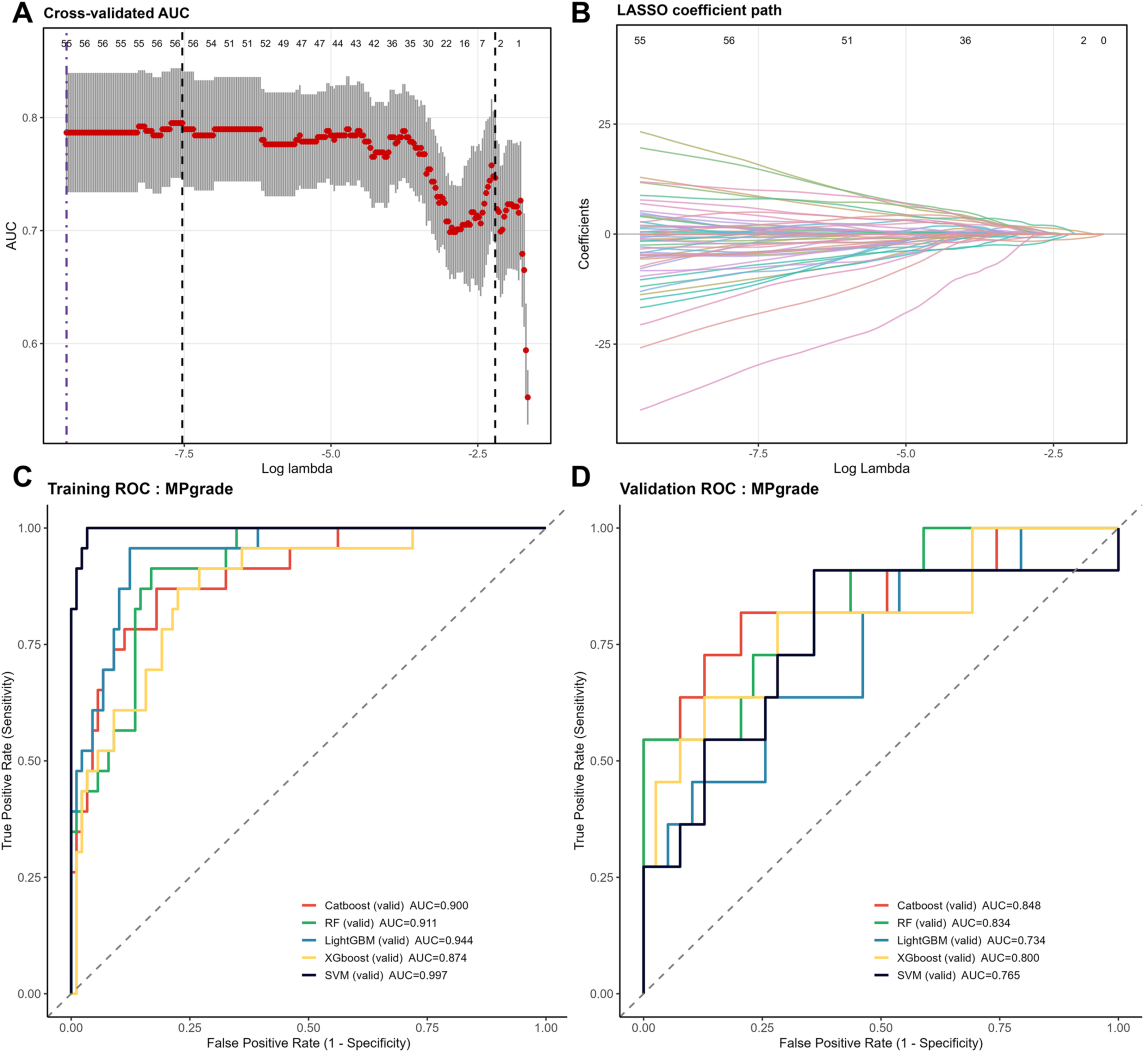
Selection process for the feature and machine learning algorithm for the nac efficacy prediction model. **(A, B)** Cross-validation curves and coefficient regularization paths from LASSO regression for feature selection; the optimal regularization parameter λ was chosen on the basis of an “acceptable AUC loss threshold”. **(C, D)** Summary of the ROC curves and AUC values obtained for the multiple machine learning models in the training set (70%) and validation set (30%).

**Figure 4:**
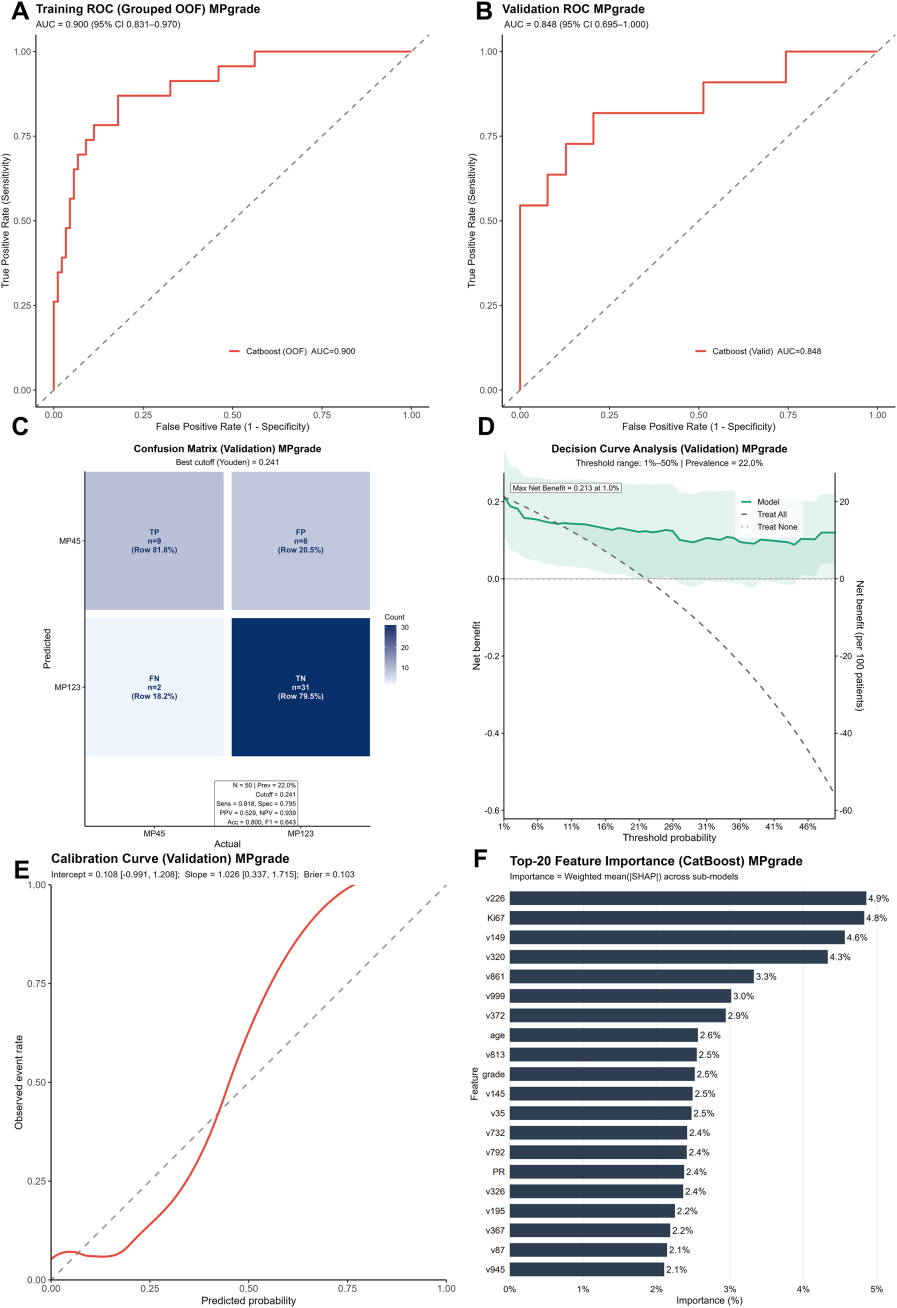
Evaluation of the predictive performance of the NAC efficacy prediction model. **(A, B)** The ROC curves and AUC values obtained for the CatBoost model in the training set (70%) and validation set (30%). **(C)** Confusion matrix yielded by the CatBoost model on the validation set. **(D)** Decision curve analysis (DCA) of the CatBoost model on the validation set. **(E)** Calibration curves of the CatBoost model in the validation set. **(F)** Feature importance ranking of the CatBoost model.

The workflow for model construction is shown in **Figure 2**. For each patient, 13 clinical variables and 1,024 pathological feature variables were extracted on the basis of the scanning and feature extraction of core needle biopsy H&E-stained histopathological slides. To mitigate the risk of model overfitting due to an excessive number of variables, least absolute shrinkage and selection operator (LASSO) regression was employed for further variable selection. In LASSO regression, the optimal set of retained variables is determined by the choice of the regularization parameter λ. We utilized 10-fold cross-validation and selected the optimal λ based on a prespecified “acceptable AUC loss threshold,” which balances model performance and the retention of potentially informative predictors; specifically, the maximum absolute decrease in the area under the curve (AUC) was set to 0.01 (**Figures 3A, B**). Ultimately, 55 variables were retained for model development, comprising six clinical variables—PR (progesterone receptor), Ki-67, glucose, BMI, age, and histological grade—and 49 pathological feature variables. Multiple machine learning models, including CatBoost, random forest, LightGBM, XGBoost, and support vector machine (SVM), were subsequently developed, and their predictive performances were assessed on both the training and validation cohorts (**Figures 3C, D**; **Table 2**). Among these models, CatBoost demonstrated the best performance in the validation set, with an AUC of 0.848, a sensitivity of 0.818, a Brier score of 0.103, and a strong specificity of 0.795, all of which indicate excellent predictive accuracy and stability (**Figures 4A, B**). Further assessment revealed that the confusion matrix for CatBoost contained a large number of true positives and true negatives (**Figure 4C**). Decision curve analysis (DCA) revealed that the net benefit curve of the model exceeded that of both the “treat all” and “treat none” strategies (representing scenarios where all or none of the patients received NAC, respectively), further supporting its clinical utility (**Figure 4D**). In addition, calibration curve analysis demonstrated good concordance between the predicted and observed probabilities of NAC response (**Figure 4E**). On the basis of these comprehensive assessments, the CatBoost model was selected as the final predictive model. Finally, analysis of the top 20 most important variables included in the CatBoost model revealed that Ki-67 ranked as the second most influential predictor. Other clinical variables in the top 20 included age, histological grade, and PR, which is consistent with our previous analysis of baseline patient characteristics (**Figure 4F**). The remaining top predictors were pathological feature variables, highlighting the critical role of pathology-derived features in the prediction of NAC response.

**Table 2:** Summary of the AUC values and predictive efficacy metrics obtained for the multiple machine learning models in the training set (70%) and validation set (30%).

Training set						
Models	AUC	Sensitivity	Specificity	Accuracy	Threshold	Brier
Catboost	0.900	0.870	0.820	0.830	0.126	0.113
RF	0.911	0.913	0.831	0.848	0.242	0.113
lightGBM	0.944	0.957	0.876	0.893	0.083	0.082
Xgboost	0.874	0.870	0.775	0.795	0.210	0.127
SVM	0.997	1.00	0.966	0.973	0.331	0.033
Validation set						
Models	AUC	Sensitivity	Specificity	Accuracy	Threshold	Brier
Catboost	0.848	0.818	0.795	0.8	0.241	0.103
RF	0.834	0.545	0.949	0.86	0.407	0.11
lightGBM	0.734	0.909	0.462	0.56	0.117	0.155
Xgboost	0.8	0.818	0.718	0.74	0.21	0.122
SVM	0.765	0.545	0.846	0.78	0.29	0.206

### 3.3 Construction and validation of the recurrence prediction model

**Figure 5:**
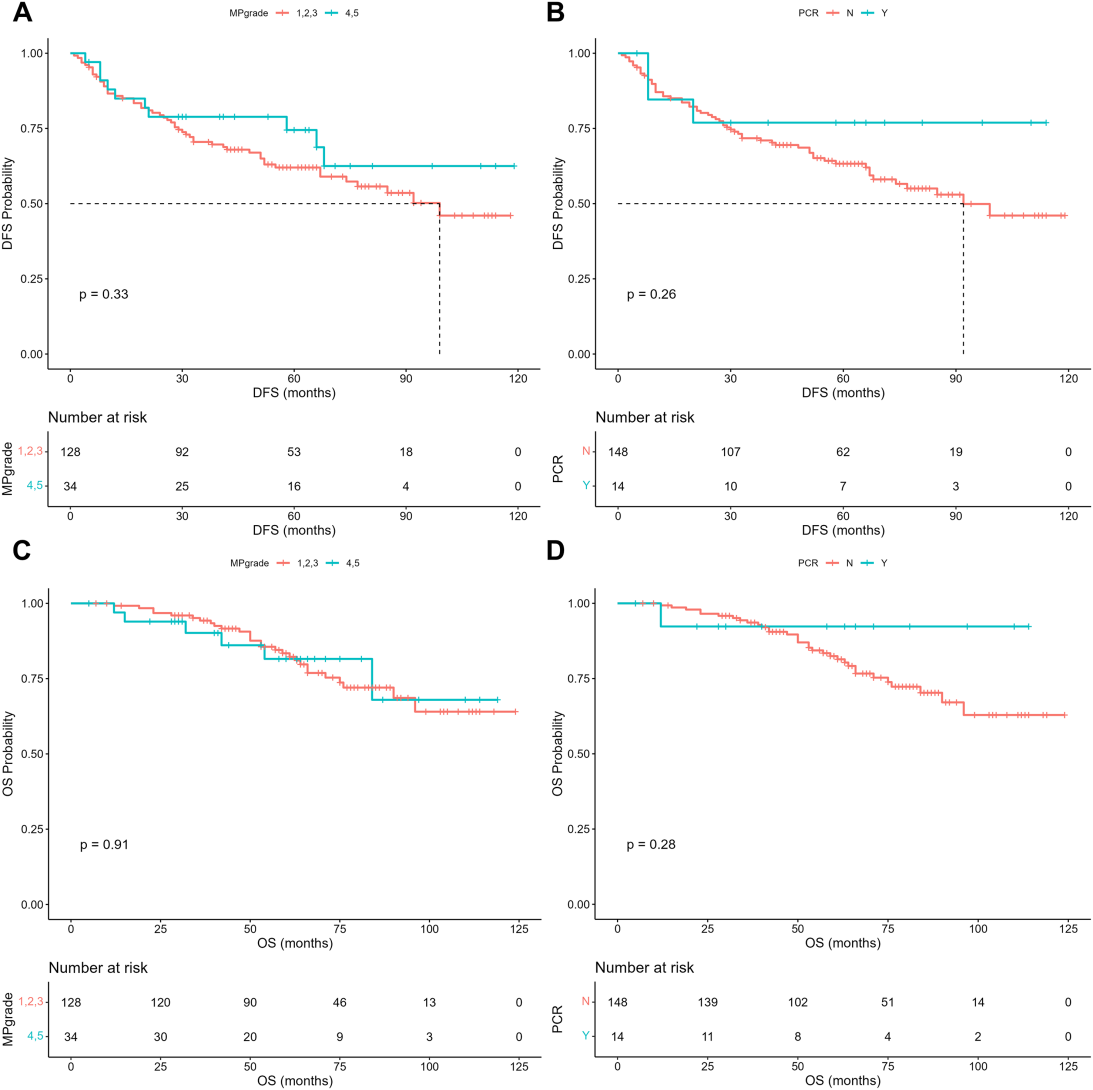
Survival curves stratified by NAC efficacy groups. **(A)** Disease-free survival curves stratified according to whether patients achieved MP4.5. **(B)** Disease-free survival curves stratified according to whether patients achieved pCR. **(C)** Overall survival curves stratified according to whether patients achieved MP4.5. **(D)** Overall survival curves stratified according to whether patients achieved pCR. Log-rank tests were used to compare two overall survival curves.

**Figure 6:**
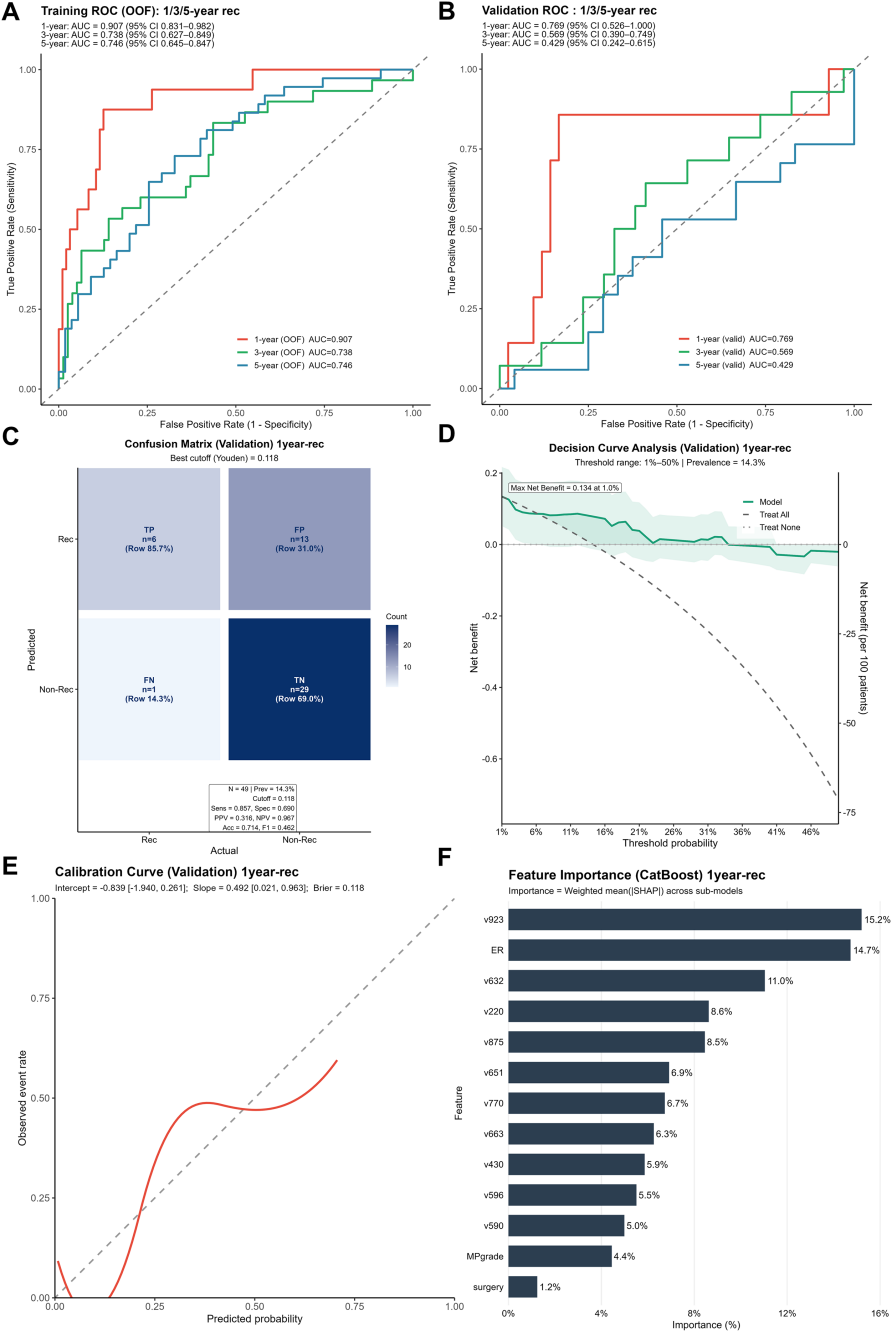
Evaluation of the predictive performance of the recurrence prediction model. **(A, B)** The ROC curves and AUC values obtained for the CatBoost model in the training set (70%) and validation set (30%). **(C)** Confusion matrix yielded by the CatBoost model on the validation set. **(D)** Decision curve analysis (DCA) of the CatBoost model on the validation set. **(E)** Calibration curves of the CatBoost model in the validation set. **(F)** Feature importance ranking of the CatBoost model.

For patients with HR-positive, HER2-negative breast cancer, the efficacy of neoadjuvant chemotherapy (NAC) not only plays a crucial role in optimizing surgical strategies but also draws considerable attention to its association with patient prognosis. Therefore, we divided the patients into two groups according to whether their MP grade reached 4 or 5 and whether a pCR was achieved. On the basis of the follow-up data, we constructed Kaplan–Meier (KM) survival plots for each group, with a median follow-up period of 66 months. The results indicate that regardless of stratification by MP grade or pCR status, there was no significant difference in recurrence status or survival outcomes between the two groups (**Figures 5A-D**). To further investigate potential risk factors influencing prognosis in this population, we performed both univariable and multivariable Cox proportional hazards regression analyses (**Table 3**; **Supplementary Table S1**). The results revealed that extreme age groups at diagnosis (20–30 years, hazard ratio [HR]=6.32, p=0.017; 71–80 years, HR = 7.29, p=0.045), clinical N stage III (HR = 2.37, p=0.033), low PR expression (0–25%; HR = 2.60, p=0.049), and MP grade 2 (HR = 4.07, p=0.003) were associated with an increased risk of recurrence. Conversely, clinical T stage I before NAC (HR = 0.20, p=0.031) and pathological T stage 0 after NAC (HR = 0.14, p=0.015) were protective factors against recurrence. Similarly, in the multivariable Cox regression analysis of survival outcomes, we observed consistent findings (**Table 4**; **Supplementary Table S2**). Low age at diagnosis (20–30 years; HR = 9.52, p=0.043), low PR expression (0–25%; HR = 6.23, p=0.027), high Ki-67 expression (51–75%; HR = 6.35, p=0.045), and MP grade 1 (HR = 12.35, p=0.021) or 2 (HR = 11.82, p=0.001) were associated with a higher risk of mortality. In contrast, compared with pathological T stage 1 disease, pathological T stage 0 disease after NAC was associated with a lower risk of mortality (HR = 0.02, p=0.026). Although these findings revealed certain risk factors linked to prognosis in HR-positive, HER2-negative breast cancer patients receiving NAC, no single variable emerged as a strong independent prognostic indicator for this population. Therefore, we next investigated the utility of machine learning models incorporating pathomic features for predicting patient prognosis. Following the previously established modeling strategy, we developed CatBoost models to predict recurrence at 1, 3, and 5 years after treatment. The results demonstrated that the model performed well in predicting 1-year recurrence, achieving an area under the curve (AUC) of 0.907 in the training set and 0.769 in the validation set, with a sensitivity of 0.857 (**Figures 6A-C**). Furthermore, decision curve analysis (DCA) demonstrated that the net benefit of the model exceeded that of both the “treat all” and “treat none” strategies (**Figure 6D**). In addition, the calibration curve showed good agreement between the predicted and observed recurrence probabilities (**Figure 6E**). Collectively, these findings support the potential clinical applicability of the recurrence prediction model. We also ranked the feature importance within the CatBoost model and found that the top-ranked variable was a pathomics feature. Among the top ten most important features, all except ER expression were pathomic variables (**Figure 6F**). These findings indicate that pathomic features may have greater potential than traditional clinical parameters for predicting recurrence and identifying high-risk subgroups among HR-positive, HER2-negative breast cancer patients.

**Table 3:** Multivariate Cox regression analysis for disease-free survival.

Variable	Group	HR (DFS)	CI 95	P value
Age	20-30	6.32	(1.38-28.96)	0.017
	31-40	2.11	(0.74-6.02)	0.163
	41-50	1.00	Reference	Reference
	51-60	1.39	(0.48-4.06)	0.541
	61-70	1.22	(0.36-4.18)	0.751
	71-80	7.29	(1.04-50.97)	0.045
cT stage	1	0.20	(0.05-0.86)	0.031
	2	1.00	Reference	Reference
	3	2.05	(0.69-6.06)	0.196
	4	0.94	(0.38-2.35)	0.898
cN stage	0	0.88	(0.19-4.1)	0.869
	1	0.89	(0.36-2.2)	0.808
	2	1.00	Reference	Reference
	3	2.37	(1.07-5.23)	0.033
Surgery	BCS	2.58	(0.87-7.69)	0.089
	Mastectomy	1.00	Reference	Reference
ER	0-0.25	0.76	(0.21-2.77)	0.679
	0.26-0.5	2.67	(0.7-10.16)	0.15
	0.51-0.75	1.92	(0.81-4.55)	0.14
	0.76-1	1.00	Reference	Reference
PR	0-0.25	2.60	(1-6.76)	0.049
	0.26-0.5	1.62	(0.6-4.37)	0.338
	0.51-0.75	1.94	(0.78-4.8)	0.152
	0.76-1	1.00	Reference	Reference
Ki67	0-0.25	1.00	Reference	Reference
	0.26-0.5	1.49	(0.71-3.1)	0.289
	0.51-0.75	0.80	(0.23-2.76)	0.727
	0.76-1	0.92	(0.2-4.28)	0.918
Grade	1	0.00	(0-Inf)	0.996
	2	1.00	Reference	Reference
	3	1.74	(0.66-4.57)	0.261
Chemotherapy regimen	AT-based	1.00	Reference	Reference
	A-based	0.00	(0-Inf)	0.999
	T-based	1.30	(0.51-3.32)	0.589
ypT stage	0	0.14	(0.03-0.69)	0.015
	1	1.00	Reference	Reference
	2	0.74	(0.36-1.55)	0.428
	3	1.76	(0.49-6.38)	0.386
	4	5.05	(0.94-27.15)	0.059
ypN stage	0	1.08	(0.27-4.26)	0.917
	1	0.62	(0.24-1.6)	0.322
	2	0.47	(0.16-1.33)	0.154
	3	1.00	Reference	Reference
Menopausal status	No	1.00	Reference	Reference
	Yes	0.73	(0.25-2.12)	0.569
Family history	No	1.00	Reference	Reference
	Yes	0.57	(0.28-1.15)	0.114
BMI	<18.4	0.00	(0-Inf)	0.997
	18.5-24.9	1.00	Reference	Reference
	25-29.9	1.01	(0.49-2.06)	0.981
	>30	0.60	(0.17-2.09)	0.423
LDL-C	<1.88	1.05	(0.28-3.94)	0.948
	1.89-4.21	1.00	Reference	Reference
	>4.22	0.90	(0.32-2.54)	0.848
TG	0-1.7	1.00	Reference	Reference
	>1.8	1.22	(0.51-2.9)	0.651
Glucose	3.9-6.1	1.00	Reference	Reference
	>6.2	0.50	(0.18-1.33)	0.164
MPgrade	1	1.67	(0.44-6.37)	0.452
	2	4.07	(1.64-10.11)	0.003
	3	1.00	Reference	Reference
	4	1.39	(0.36-5.44)	0.635
	5	0.00	(0-Inf)	0.999
PCR	No	1.00	Reference	Reference
	Yes	0.60	(0.05-7.56)	0.693

**Table 4:** Multivariate Cox regression analysis for overall survival of patients.

Variable	Group	HR (OS)	CI 95	P value
Age	20-30	9.52	(1.07-84.81)	0.043
	31-40	3.61	(0.62-20.95)	0.152
	41-50	1.00	Reference	Reference
	51-60	1.83	(0.38-8.84)	0.452
	61-70	0.63	(0.05-7.26)	0.711
	71-80	9.71	(0.39-244.12)	0.167
cT stage	1	0.19	(0.02-2.13)	0.178
	2	1.00	Reference	Reference
	3	3.41	(0.78-14.84)	0.102
	4	1.27	(0.26-6.19)	0.767
cN stage	0	0.00	(0-Inf)	0.998
	1	2.17	(0.57-8.19)	0.254
	2	1.00	Reference	Reference
	3	2.56	(0.72-9.15)	0.148
Surgery	BCS	0.80	(0.09-6.88)	0.841
	Mastectomy	1.00	Reference	Reference
ER	0-0.25	2.29	(0.39-13.57)	0.36
	0.26-0.5	1.65	(0.16-17.36)	0.676
	0.51-0.75	1.97	(0.5-7.85)	0.335
	0.76-1	1.00	Reference	Reference
PR	0-0.25	6.23	(1.23-31.53)	0.027
	0.26-0.5	2.84	(0.64-12.54)	0.167
	0.51-0.75	1.14	(0.17-7.66)	0.896
	0.76-1	1.00	Reference	Reference
Ki67	0-0.25	1.00	Reference	Reference
	0.26-0.5	1.30	(0.37-4.61)	0.685
	0.51-0.75	6.35	(1.04-38.7)	0.045
	0.76-1	6.30	(0.33-120.65)	0.222
Grade	1	0.00	(0-Inf)	0.998
	2	1.00	Reference	Reference
	3	0.73	(0.17-3.02)	0.66
Chemotherapy regimen	AT-based	1.00	Reference	Reference
	A-based	0.00	(0-Inf)	0.999
	T-based	0.36	(0.05-2.44)	0.294
ypT stage	0	0.02	(0-0.62)	0.026
	1	1.00	Reference	Reference
	2	0.37	(0.11-1.22)	0.102
	3	0.15	(0.01-1.71)	0.127
	4	2.35	(0.12-46.86)	0.576
ypN stage	0	0.14	(0.01-2.46)	0.179
	1	0.51	(0.1-2.57)	0.415
	2	0.27	(0.04-1.97)	0.195
	3	1.00	Reference	Reference
Menopausal status	No	1.00	Reference	Reference
	Yes	0.20	(0.03-1.13)	0.068
Family History	No	1.00	Reference	Reference
	Yes	0.38	(0.11-1.31)	0.125
BMI	<18.4	0.00	(0-Inf)	0.999
	18.5-24.9	1.00	Reference	Reference
	25-29.9	1.40	(0.45-4.36)	0.564
	>30	0.75	(0.09-6.63)	0.799
LDL-C	<1.88	0.21	(0.01-5.99)	0.363
	1.89-4.21	1.00	Reference	Reference
	>4.22	2.26	(0.41-12.63)	0.352
TG	0-1.7	1.00	Reference	Reference
	>1.8	0.70	(0.19-2.59)	0.599
Glucose	3.9-6.1	1.00	Reference	Reference
	>6.2	0.90	(0.18-4.54)	0.9
MPgrade	1	12.35	(1.47-103.76)	0.021
	2	11.82	(2.61-53.65)	0.001
	3	1.00	Reference	Reference
	4	1.25	(0.15-10.46)	0.836
	5	0.03	(0-Inf)	1
PCR	No	1.00	Reference	Reference
	Yes	3.31e+10	(0-Inf)	0.999

## 4 Discussion

In this study, we developed and validated a machine learning model based on the CatBoost algorithm to predict the efficacy of neoadjuvant chemotherapy (NAC) in patients with HR-positive, HER2-negative breast cancer. Furthermore, we investigated prognostic risk factors and evaluated the model performance in predicting recurrence in this patient population. Our findings demonstrate that by integrating pathomic features from pre-NAC biopsy histopathological slides with clinical data, the model achieves high accuracy in both treatment response prediction and recurrence risk stratification, highlighting its promising potential for clinical application.

Given the substantial clinical importance of predicting the response to NAC, numerous previous studies have focused on this topic. Liu et al. (18) and Wang et al. (19) developed predictive models based on patients’ clinical characteristics combined with MRI-based radiomics features to estimate NAC efficacy in HR-positive/HER2-negative breast cancer, achieving favorable predictive performance, with AUCs of 0.787 and 0.810 in the validation cohorts respectively, slightly lower than that observed in the present study (AUC = 0.848). However, unlike the current pathomics based approach, most radiomics driven models rely on manual delineation of regions of interest (ROIs), a process that is labor intensive and time consuming, thereby limiting their feasibility and scalability in routine clinical practice. In addition, li et al. (16) constructed a machine learning model based on pathomics features to predict NAC response across all breast cancer subtypes, achieving an AUC of 0.830 in the validation set. However, that study used CellProfiler to extract pathology features, whereas our study used the UNI algorithm, which can automatically segment and extract feature information from pathology slides. CellProfiler requires pathologists to preselect screenshots with the highest tumor cell content from segmented WSIs for subsequent feature extraction, which increases labor costs. In addition, unlike li et al., who chose a single SVM as the predictive algorithm, our study compared five different machine learning algorithms and built ensemble models to further improve predictive performance; this may explain why our study, focusing on the HR-positive, HER2-negative subgroup, was still able to achieve a higher AUC.

Unlike HER2-overexpressing or triple-negative breast cancer (TNBC), HR-positive, HER2-negative breast cancer is less sensitive to neoadjuvant chemotherapy (NAC). Previous studies have reported that the pCR rate in this population ranges from approximately 0–18% (20, 21). Consistent with these findings, our cohort had a pCR rate of 8.6%, with only 14 out of 162 patients achieving pCR following NAC. The lower sensitivity to chemotherapy in this subtype may be attributed to hormone dependency, lower cellular proliferation rates, and distinctive mutational characteristics of tumor cells (22). One of the goals of NAC is to reduce tumor volume so that patients who would otherwise require mastectomy may become eligible for breast-conserving surgery (BCS), thereby minimizing surgical trauma and improving quality of life (23), for these patients, achieving pCR is not always necessary for surgical de-escalation (24). For this reason, we selected Miller–Payne (MP) grade as the primary endpoint for model prediction, with grades 4 and 5 indicating substantial tumor regression (17). In our cohort, the proportion of patients who ultimately underwent BCS was significantly greater among those with MP grades 4 or 5 than among those with grades 1, 2, or 3 (20.6% vs. 4.7%, P = 0.007). Previous research has also demonstrated that NAC increases the rate of breast conservation in this subgroup of patients (25–29). Despite this benefit, only a small percentage of HR-positive, HER2-negative patients achieve high MP grades after NAC, which highlights the need to identify patients who are unlikely to benefit from chemotherapy in advance and avoid subjecting them to unnecessary toxicity. Currently, there are few robust tools for predicting NAC response in this population. Most prior models focus primarily on conventional clinical and molecular pathological markers, including hormone receptor status, the Ki-67 index, clinical tumor stage, and histological grade (8, 9, 30). In our study, we observed significant differences in these features between NAC-sensitive and NAC-insensitive patients, with the sensitive group generally being younger, exhibiting lower expression of estrogen receptor (ER) and progesterone receptor (PR), and having higher values for Ki-67 and histological grade. Most of these differences are molecular pathological features, suggesting that additional discriminative information may be embedded within pathological images. This rationale led us to integrate both pathomic features and clinical variables into our predictive model. Pathomic features were extracted from hematoxylin and eosin (H&E)-stained whole-slide images (WSIs) of core needle biopsy specimens obtained prior to NAC. These images are readily available in clinical practice, cost effective, and offer high potential for clinical translation (31). For feature extraction, we utilized the UNI model, which had been pretrained on large-scale cancer image databases containing more than 100,000 H&E-stained diagnostic-grade whole-slide images across 20 major tissue types, exceeding 77 terabytes in total. Through self-supervised and multitask learning, the UNI model captures complex morphological patterns and features from cancer histopathology, enabling efficient and accurate feature extraction even on new datasets (32). During the model development stage, we tested five commonly used machine learning algorithms and reported that CatBoost provided the highest accuracy and sensitivity in predicting NAC response. In future clinical applications, false-positive predictions present a greater risk, as misclassifying chemotherapy-insensitive patients as sensitive could deprive them of the opportunity for surgery. Given this consideration, CatBoost was selected as the optimal modeling strategy. As a decision tree-based algorithm, CatBoost is well suited for handling categorical and heterogeneous data, offering both robust accuracy and generalizability in medical prediction tasks (33–36). All the steps of model construction were performed exclusively within the training set to ensure that no information leakage into the validation set occurred, thereby maintaining the integrity and reliability of the prediction results. For the final prediction model, we applied SHapley Additive exPlanations (SHAP) to interpret feature importance. As anticipated, in addition to previously reported clinical predictors such as PR and Ki-67, pathomic features were predominant among the most important variables. These findings support our hypothesis and highlight the value of pathomic data in predicting NAC response in patients with breast cancer. The model development process, from biopsy slide scanning and feature extraction to variable selection and model training, is fully reproducible and presents significant promise for future clinical translation.

The relationship between the efficacy of neoadjuvant chemotherapy (NAC) and patient prognosis remains an important area of investigation. Previous studies have reported that achieving pathological complete response (pCR) after NAC is often associated with improved prognosis (37–40). However, our results indicate that for patients with HR-positive, HER2-negative breast cancer, neither achieving pCR nor attaining Miller–Payne (MP) grades 4 or 5 after NAC is associated with a reduced risk of recurrence or mortality. These findings align with reports from some previous studies, which have also shown conflicting results in this patient subgroup (11, 41, 42). Our further analyses provide a possible explanation for this phenomenon. Cox proportional hazards regression revealed risk factors for worse prognosis, including younger age at diagnosis, low progesterone receptor expression, and a high Ki-67 index. However, these factors are also associated with a better response to chemotherapy. Thus, the prognostic benefit conferred by the chemotherapy response is offset by the higher baseline risk associated with more aggressive tumor biology, leading to the observation that NAC responders and nonresponders have similar long-term outcomes in this patient population. In light of this, the identification of high-risk patients for intensified therapy is essential to reduce the risk of recurrence. Our results demonstrate that machine learning models integrating pathomics and clinical information strongly predict 1-year recurrence, but these models do not accurately predict 3- or 5-year recurrence. This may be because recurrence within 1 year represents a very high degree of malignancy of the tumor, with certain identifiable characteristics at the pathological level. However, recurrence after a longer period is influenced by more diverse factors, and relying solely on information from the tumor itself may not be sufficient for accurate prediction. We also investigated the application of our models for survival prediction. Owing to the low number of deaths within 1 year, we developed models to predict 3-year and 5-year survival. Although the 3-year survival prediction model achieved a favorable area under the curve (AUC) of 0.770 in the validation set, decision curve analysis revealed no clinical net benefit for patient management (**Supplementary Figures S2A, B, D**). Analysis of the confusion matrix suggested that this is attributable to the overall favorable prognosis of this subtype, with a 3-year mortality rate of only 6.7%; assigning the majority of patients to the “survival” category yields high specificity (0.952) but fails to provide meaningful risk stratification despite an apparently good AUC (**Supplementary Figure S2C**).

The novelty of the present study lies in the development of a subtype specific, fully automated model for predicting NAC response in HR-positive,HER2-negative breast cancer, based on a rigorous and reproducible analytical pipeline. In addition, we investigated prognostic risk factors in this patient subgroup and evaluated the performance of the proposed model in predicting disease recurrence, thereby extending previous machine learning–based pathology research. Nevertheless, this study has several limitations: First, our data were collected from a single-center retrospective cohort and lack external validation. Despite strict inclusion criteria to ensure data quality and the use of ensemble modeling to increase model robustness and generalizability, larger prospective cohorts and the incorporation of external validation datasets will be needed in future work to improve the model reliability and predictive accuracy. Second, in this study, pathomic features were extracted using the UNI model. While UNI enables efficient and accurate feature extraction, as a patch-level feature extractor, it generates encoded feature vectors rather than manually engineered morphological features, such as nuclear shape or tissue architecture (43). Although this abstract representation can capture complex pathological patterns, it does not allow direct mapping back to the original image to form interpretable visual markers, for example, overlaying heatmaps for intuitive pathological localization. As a result, the model lacks straightforward pathological interpretability. In future research, building on our existing work, we will continue to develop interpretable predictive models to further improve the model’s predictive performance.

In conclusion, we developed a CatBoost-based machine learning model that integrates pathomics and clinical information to predict the efficacy of NAC in HR-positive, HER2-negative breast cancer and demonstrated its robust predictive performance. Furthermore, our pathological model predicted early recurrence in HR-positive, HER2-negative breast cancer patients, thus could provide better guidance for clinical practice when deciding whether to perform surgery first or NAC.

## Data Availability

The raw data supporting the conclusions of this article will be made available by the authors, without undue reservation.
