# Supplementary material for: Development and validation of a pathomics-driven machine learning model for individualized prediction of neoadjuvant chemotherapy response and early recurrence in HR-positive, HER2-negative breast cancer
Source: Front Oncol. 2026 Feb 23;16:1770037. doi: 10.3389/fonc.2026.1770037 (PMC12967959; doi:10.3389/fonc.2026.1770037)
Supplement: Supplementary file 1 [file DataSheet1.docx]

Supplementary Material

# Supplementary Figures and Tables

## Supplementary Figures


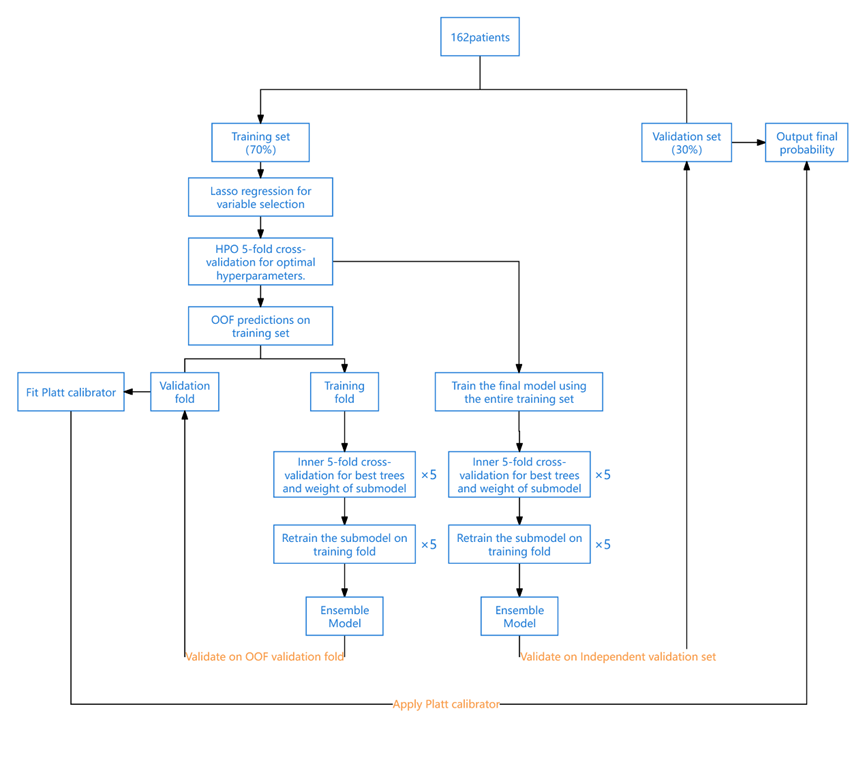


**Supplementary Figure 1.** The construction and validation process of the model.


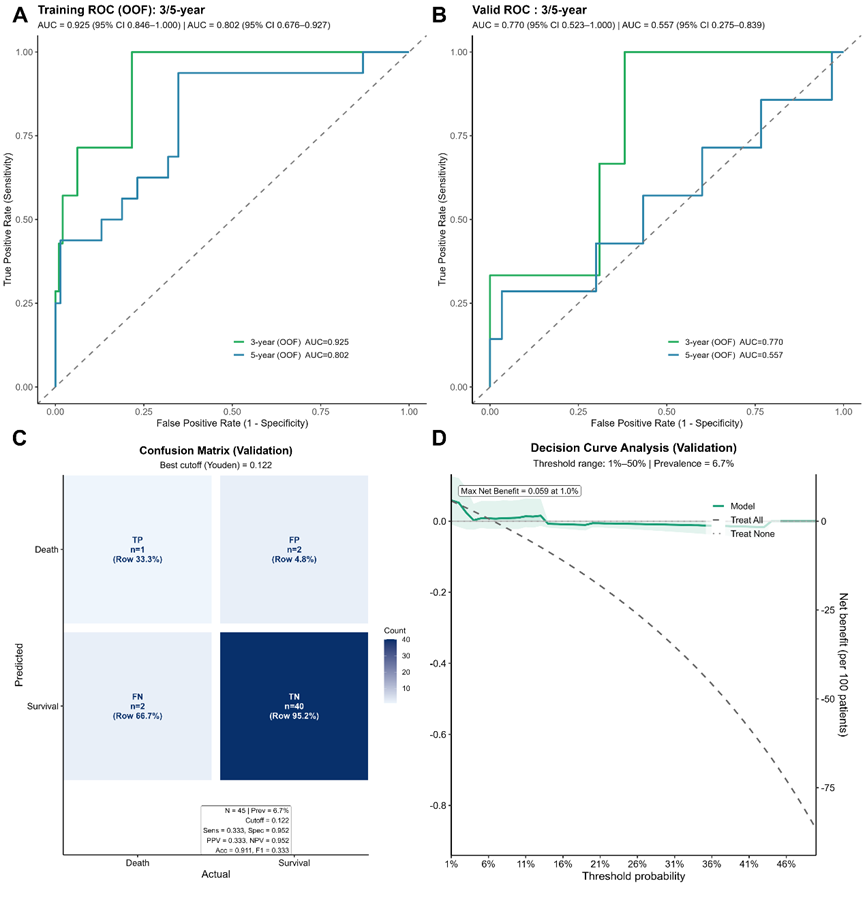


**Supplementary Figure 2.** Evaluation of the Predictive Performance of the Survival Prediction Model. (A, B) The ROC curves and AUC values obtained for the CatBoost model in the training set (70%) and valida-tion set (30%). (C) Confusion matrix yielded by the CatBoost model on the validation set. (D) Decision curve analysis (DCA) of the CatBoost model on the validation set.

## Supplementary Tables

**Supplemental Table.1** Univariate Cox regression analysis for disease-free survival of patients

| Variable | Group | HR(DFS) | CI 95 | P value |
| --- | --- | --- | --- | --- |
| Age | 20-30 | 1.55 | (0.44-5.42) | 0.491 |
|  | 31-40 | 2.48 | (1.17-5.23) | 0.017 |
|  | 41-50 | 1.00 | Reference | Reference |
|  | 51-60 | 1.50 | (0.74-3.04) | 0.261 |
|  | 61-70 | 1.30 | (0.6-2.8) | 0.511 |
|  | 71-80 | 1.63 | (0.47-5.68) | 0.445 |
| cT stage | 1 | 0.36 | (0.11-1.18) | 0.092 |
|  | 2 | 1.00 | Reference | Reference |
|  | 3 | 1.09 | (0.52-2.25) | 0.824 |
|  | 4 | 0.82 | (0.44-1.52) | 0.528 |
| cN stage | 0 | 0.66 | (0.23-1.92) | 0.446 |
|  | 1 | 0.81 | (0.41-1.58) | 0.527 |
|  | 2 | 1.00 | Reference | Reference |
|  | 3 | 1.62 | (0.9-2.92) | 0.106 |
| Surgery | BCS | 1.41 | (0.64-3.09) | 0.398 |
|  | Mastectomy | 1.00 | Reference | Reference |
| ER | 0-0.25 | 1.02 | (0.43-2.41) | 0.959 |
|  | 0.26-0.5 | 5.45 | (2.11-14.11) | <0.001 |
|  | 0.51-0.75 | 2.25 | (1.16-4.39) | 0.017 |
|  | 0.76-1 | 1.00 | Reference | Reference |
| PR | 0-0.25 | 2.60 | (1.34-5.04) | 0.005 |
|  | 0.26-0.5 | 1.96 | (0.92-4.18) | 0.08 |
|  | 0.51-0.75 | 1.99 | (0.92-4.3) | 0.081 |
|  | 0.76-1 | 1.00 | Reference | Reference |
| Ki67 | 0-0.25 | 1.00 | Reference | Reference |
|  | 0.26-0.5 | 1.44 | (0.84-2.49) | 0.188 |
|  | 0.51-0.75 | 1.75 | (0.75-4.09) | 0.195 |
|  | 0.76-1 | 1.50 | (0.52-4.34) | 0.453 |
| Grade | 1 | 0.00 | (0-Inf) | 0.996 |
|  | 2 | 1.00 | Reference | Reference |
|  | 3 | 1.50 | (0.81-2.76) | 0.195 |
| Chemotherapy regimen | AT-based | 1.00 | Reference | Reference |
|  | A-based | 0.00 | (0-Inf) | 0.995 |
|  | T-based | 0.65 | (0.31-1.36) | 0.253 |
| ypT stage | 0 | 0.72 | (0.27-1.89) | 0.507 |
|  | 1 | 1.00 | Reference | Reference |
|  | 2 | 0.95 | (0.54-1.66) | 0.856 |
|  | 3 | 1.52 | (0.62-3.74) | 0.357 |
|  | 4 | 1.65 | (0.5-5.5) | 0.412 |
| ypN stage | 0 | 0.47 | (0.22-1) | 0.05 |
|  | 1 | 0.60 | (0.32-1.13) | 0.114 |
|  | 2 | 0.33 | (0.16-0.68) | 0.003 |
|  | 3 | 1.00 | Reference | Reference |
| Menopausal status | NO | 1.00 | Reference | Reference |
|  | YES | 0.97 | (0.59-1.59) | 0.893 |
| Family history | NO | 1.00 | Reference | Reference |
|  | YES | 0.81 | (0.46-1.41) | 0.457 |
| BMI | 0-18.4 | 0.00 | (0-Inf) | 0.995 |
|  | 18.5-24.9 | 1.00 | Reference | Reference |
|  | 25-29.9 | 0.88 | (0.51-1.49) | 0.626 |
|  | 30-43 | 0.83 | (0.35-1.97) | 0.672 |
| LDL-C | 0-1.88 | 0.84 | (0.3-2.31) | 0.73 |
|  | 1.89-4.21 | 1.00 | Reference | Reference |
|  | 4.22-6 | 0.90 | (0.38-2.09) | 0.801 |
| TG | 0-1.7 | 1.00 | Reference | Reference |
|  | 1.8-7 | 0.93 | (0.49-1.74) | 0.811 |
| Glucose | 3.9-6.1 | 1.00 | Reference | Reference |
|  | 6.2-18 | 0.71 | (0.34-1.49) | 0.368 |
| MPgrade | 1 | 2.32 | (0.88-6.12) | 0.088 |
|  | 2 | 1.51 | (0.86-2.66) | 0.155 |
|  | 3 | 1.00 | Reference | Reference |
|  | 4 | 1.08 | (0.41-2.86) | 0.869 |
|  | 5 | 0.78 | (0.3-2.06) | 0.617 |
| PCR | NO | 1.00 | Reference | Reference |
|  | YES | 0.52 | (0.16-1.66) | 0.269 |

**Supplemental Figure. 2** Univariate Cox regression analysis for overall survival of patients

| Variable | Group | HR(OS) | CI 95 | P value |
| --- | --- | --- | --- | --- |
| Age | 20-30 | 1.78 | (0.37-8.49) | 0.469 |
|  | 31-40 | 2.90 | (1.1-7.61) | 0.031 |
|  | 41-50 | 1.00 | Reference | Reference |
|  | 51-60 | 1.65 | (0.65-4.18) | 0.291 |
|  | 61-70 | 0.54 | (0.14-2.06) | 0.369 |
|  | 71-80 | 1.67 | (0.35-7.87) | 0.517 |
| cT stage | 1 | 0.26 | (0.03-1.92) | 0.185 |
|  | 2 | 1.00 | Reference | Reference |
|  | 3 | 1.42 | (0.57-3.56) | 0.454 |
|  | 4 | 0.93 | (0.41-2.12) | 0.861 |
| cN stage | 0 | 0.00 | (0-Inf) | 0.997 |
|  | 1 | 1.05 | (0.45-2.44) | 0.907 |
|  | 2 | 1.00 | Reference | Reference |
|  | 3 | 1.53 | (0.69-3.42) | 0.297 |
| Surgery | BCS | 0.69 | (0.17-2.9) | 0.617 |
|  | Mastectomy | 1.00 | Reference | Reference |
| ER | 0-0.25 | 2.23 | (0.82-6.03) | 0.115 |
|  | 0.26-0.5 | 8.06 | (2.69-24.19) | <0.001 |
|  | 0.51-0.75 | 3.02 | (1.26-7.25) | 0.013 |
|  | 0.76-1 | 1.00 | Reference | Reference |
| PR | 0-0.25 | 3.84 | (1.47-10.03) | 0.006 |
|  | 0.26-0.5 | 3.42 | (1.24-9.42) | 0.017 |
|  | 0.51-0.75 | 1.43 | (0.4-5.1) | 0.578 |
|  | 0.76-1 | 1.00 | Reference | Reference |
| Ki67 | 0-0.25 | 1.00 | Reference | Reference |
|  | 0.26-0.5 | 1.28 | (0.58-2.8) | 0.54 |
|  | 0.51-0.75 | 3.61 | (1.41-9.24) | 0.007 |
|  | 0.76-1 | 1.57 | (0.35-7.03) | 0.556 |
| Grade | 1 | 0.00 | (0-Inf) | 0.997 |
|  | 2 | 1.00 | Reference | Reference |
|  | 3 | 1.78 | (0.8-3.93) | 0.156 |
| Chemotherapy regimen | AT-based | 1.00 | Reference | Reference |
|  | A-based | 0.00 | (0-Inf) | 0.997 |
|  | T-based | 0.30 | (0.07-1.23) | 0.095 |
| ypT stage | 0 | 0.22 | (0.03-1.68) | 0.144 |
|  | 1 | 1.00 | Reference | Reference |
|  | 2 | 0.62 | (0.29-1.32) | 0.214 |
|  | 3 | 0.89 | (0.26-3.08) | 0.86 |
|  | 4 | 1.78 | (0.41-7.77) | 0.445 |
| ypN stage | 0 | 0.22 | (0.05-0.95) | 0.042 |
|  | 1 | 0.76 | (0.34-1.72) | 0.51 |
|  | 2 | 0.50 | (0.21-1.22) | 0.129 |
|  | 3 | 1.00 | Reference | Reference |
| Menopausal status | NO | 1.00 | Reference | Reference |
|  | YES | 0.63 | (0.31-1.28) | 0.202 |
| Family history | NO | 1.00 | Reference | Reference |
|  | YES | 0.73 | (0.34-1.56) | 0.415 |
| BMI | 0-18.4 | 0.00 | (0-Inf) | 0.997 |
|  | 18.5-24.9 | 1.00 | Reference | Reference |
|  | 25-29.9 | 0.93 | (0.46-1.89) | 0.847 |
|  | 30-43 | 0.48 | (0.11-2.08) | 0.331 |
| LDL-C | 0-1.88 | 0.41 | (0.06-3.02) | 0.382 |
|  | 1.89-4.21 | 1.00 | Reference | Reference |
|  | 4.22-6 | 1.19 | (0.42-3.39) | 0.749 |
| TG | 0-1.7 | 1.00 | Reference | Reference |
|  | 1.8-7 | 0.90 | (0.37-2.18) | 0.822 |
| Glucose | 3.9-6.1 | 1.00 | Reference | Reference |
|  | 6.2-18 | 0.73 | (0.26-2.06) | 0.547 |
| MPgrade | 1 | 2.02 | (0.56-7.27) | 0.28 |
|  | 2 | 1.68 | (0.76-3.71) | 0.2 |
|  | 3 | 1.00 | Reference | Reference |
|  | 4 | 2.85 | (0.98-8.3) | 0.054 |
|  | 5 | 0.34 | (0.04-2.64) | 0.303 |
| PCR | NO | 1.00 | Reference | Reference |
|  | YES | 0.35 | (0.05-2.59) | 0.305 |
